# Intensity-modulated irradiation for superficial tumors by overlapping irradiation fields using intensity modulators in accelerator-based BNCT

**DOI:** 10.1093/jrr/rrac052

**Published:** 2022-09-22

**Authors:** Akinori Sasaki, Naonori Hu, Takushi Takata, Nishiki Matsubayashi, Yoshinori Sakurai, Minoru Suzuki, Hiroki Tanaka

**Affiliations:** Graduate School of Engineering, Kyoto University, Kyoto University Katsura, Kyoto Nishikyo-ku, Kyoto 615-8246, Japan; Particle Radiation Oncology Research Center, Institute for Integrated Radiation and Nuclear Science, Kyoto University, 2-Asashiro-Nishi, Kumatori-cho, Sennan-gun, Osaka 590-0494, Japan; Kansai BNCT Medical Center, Educational Foundation of Osaka Medical and Pharmaceutical University, Daigakumachi, Takatsuki, Osaka 569-0801, Japan; Particle Radiation Oncology Research Center, Institute for Integrated Radiation and Nuclear Science, Kyoto University, 2-Asashiro-Nishi, Kumatori-cho, Sennan-gun, Osaka 590-0494, Japan; Graduate School of Engineering, Kyoto University, Kyoto University Katsura, Kyoto Nishikyo-ku, Kyoto 615-8246, Japan; Particle Radiation Oncology Research Center, Institute for Integrated Radiation and Nuclear Science, Kyoto University, 2-Asashiro-Nishi, Kumatori-cho, Sennan-gun, Osaka 590-0494, Japan; Particle Radiation Oncology Research Center, Institute for Integrated Radiation and Nuclear Science, Kyoto University, 2-Asashiro-Nishi, Kumatori-cho, Sennan-gun, Osaka 590-0494, Japan; Particle Radiation Oncology Research Center, Institute for Integrated Radiation and Nuclear Science, Kyoto University, 2-Asashiro-Nishi, Kumatori-cho, Sennan-gun, Osaka 590-0494, Japan

**Keywords:** overlapping radiation fields, accelerator-based boron neutron capture therapy (BNCT), uniform thermal neutron flux, intensity-modulated irradiation

## Abstract

The distribution of the thermal neutron flux has a significant impact on the treatment efficacy. We developed an irradiation method of overlapping irradiation fields using intensity modulators for the treatment of superficial tumors with the aim of expanding the indications for accelerator-based boron neutron capture therapy (BNCT). The shape of the intensity modulator was determined and Monte Carlo simulations were carried out to determine the uniformity of the resulting thermal neutron flux distribution. The intensity modulators were then fabricated and irradiation tests were conducted, which resulted in the formation of a uniform thermal neutron flux distribution. Finally, an evaluation of the tumor dose distribution showed that when two irradiation fields overlapped, the minimum tumor dose was 27.4 Gy-eq, which was higher than the tumor control dose of 20 Gy-eq. Furthermore, it was found that the uniformity of the treatment was improved 47% as compared to the treatment that uses a single irradiation field. This clearly demonstrates the effectiveness of this technique and the possibility of expanding the indications to superficially located tumors.

## INTRODUCTION

Boron neutron capture therapy (BNCT) is a type of radiation therapy that uses charged particles emitted by a nuclear reaction between ^10^B and thermal neutrons [1, 2]. These charged particles have high linear energy transfer and a short range corresponding to approximately the diameter of cancer cells. If ^10^B is accumulated in cancer cells, BNCT can thus selectively kill cancer cells.

In recent years, neutron sources for BNCT have shifted from reactor- to accelerator-based treatment systems. Accelerator-based neutron sources produce epithermal neutrons with higher neutron energy compared to reactor-based neutron sources to treat deep-seated tumors, which facilitates the treatment of tumors that are deep.

A cyclotron-based epithermal neutron source (C-BENS) was developed for the widespread use of BNCT [3]. The world’s first clinical trials using accelerator-based neutron sources have been conducted to treat recurrent brain tumors, and head and neck cancers. Based on the results of clinical trials [4, 5], an application for approval was filed, following which an approval was granted for the manufacture and marketing of the product as a medical device for the treatment of the unresectable locally advanced or locally recurrent head and neck cancer in March 2020. In addition, BNCT has been covered by insurance since June 2020 for the above head and neck cancer within the medical administration of Japan. Thus, the number of facilities performing BNCT is expected to increase in the future. While the number of facilities is increasing, expansion of indications for skin cancer, such as malignant melanoma and superficial tumors such as angiosarcoma [6] and malignant meningioma [7], are also expected. Therefore, the development of irradiation methods for superficial tumors is desirable.

Epithermal neutrons are not suitable for treating superficial tumors because thermal neutrons have to be delivered to the tumors. However it is difficult to achieve a uniform thermal neutron distribution for tumors that spread over a wide area. Consequently, it is expected that a sufficient dose cannot be administered uniformly to the tumor. To adapt accelerator-based BNCT for superficial tumors, it is essential to develop a method capable of efficiently moderating epithermal neutrons and irradiating tumors with uniform and intense thermal neutrons.

Previous studies have shown that thermal neutrons can be uniformly irradiated in irradiation fields as small as 5 cm in diameter using a bolus placed on the patient’s body [8]. The optimization of the bolus shape is expected to be effective in achieving uniform thermal neutron and dose distribution. However, the formation of a uniform thermal neutron distribution with a single irradiation using a bolus for relatively shallow and widely spreading tumors, such as angiosarcoma and malignant meningioma is difficult. Another method consists in placing a modulator material inside the collimator to shift the thermal neutron peak to the body surface [9], but a more uniform irradiation method is desirable.

In this study, we propose a method to form various thermal neutron distributions by installing neutron intensity modulators in the collimator and irradiating uniform thermal neutrons by overlapping two irradiation fields. The treatment of superficial tumors on the scalp was assumed. The shape of the intensity modulators was designed using the treatment planning system, Simulation Environment for Radiotherapy Applications (SERA) [10], with an assumption that a superficial tumor, 10 cm in diameter and 0.1 cm in thickness is being irradiated. The combination of the intensity modulators was determined to uniformly deliver the thermal neutrons. The thermal neutron distribution contributes the most to the tumor dose distribution. Next, an intensity modulator was created and irradiation tests were conducted. The dose distribution was evaluated using SERA, proving that the intensity-moderated irradiation method using accelerator-based BNCT was effective, indicating the possibility of expanding the application.

## MATERIALS AND METHODS

### Fabrication of a head phantom

**Fig. 1:**
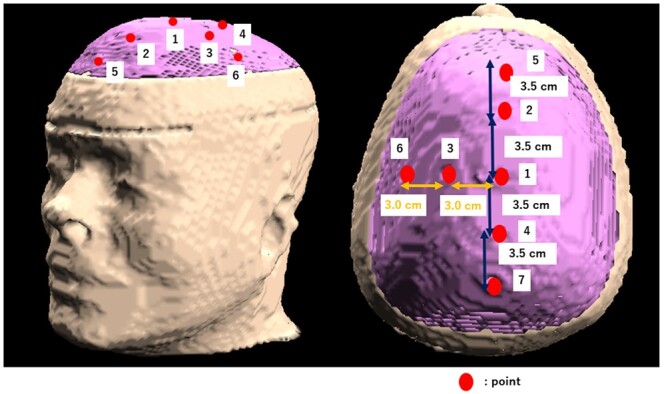
3D model of the head phantom by SERA and evaluation points.

A phantom of the head was created from computed tomography (CT) images. CT images of the head were obtained from The Cancer Imaging Archive (TCIA), a public patient image database [11–14]. From this CT image, a 3D model of the head surface was created using OsiriX surface-rendering function. To convert the 3D data for printing, 3D-CAD software Meshmixer and Fusion 360 (Autodesk, Inc.), were used. The thickness of the phantom shell was set to 0.3 cm, forming a structure that could contain water. Another structure was fabricated as a lid so that the water could be sealed for multiple uses. An O-ring was fitted into the structure to prevent water from leaking. A head phantom was fabricated with 3D data using a 3D printer AGILISTA-31103D (Keyence Corporation). CT images of this phantom were acquired at a slice thickness of 0.125 cm using ECLOS (Hitachi Medical Corporation) and imported into SERA. The SERA source data were normalized by the measured thermal neutron flux distribution in a square water phantom. Figure 1 shows the 3D model of the head phantom. Some evaluation points were established to conduct a comparison between the irradiation test and SERA calculation. Because the irradiation was from the vertex direction, it was symmetrical. Hence, there were seven evaluation points: one at the center of the irradiation, two on the left side, and two on the front and back sides with reference to Fig. 1. For the left side, the distance between the center point1–point3 and point3–point6 was 3 cm, whereas the distance between each point on the front and back sides were also set to 3 cm.

### Determination of the shape of the intensity modulators

Based on the assumption of superficial scalp tumor treatment made here, head irradiation of a superficial tumor, 10 cm in diameter and 0.1 cm in thickness, arising on the vertex using C-BENS was performed. Other than intensity modulators, the irradiation setup remained unchanged including the irradiation angle and patient position settings. Polyethylene (PE) and PE loaded with lithium fluoride (LiF-PE), which contained ^6^Li in its natural abundance ratio 7.5%, were used as intensity modulators. Their respective densities are approximately 0.95 g/cm^3^ and 1.44 g/cm^3^. Also, for LiF-PE, the weight ratio of LiF is 53%. The current treatment protocol with accelerator-based BNCT imposes that irradiation must be completed within one hour from the perspective of continuous administration of boron during irradiation [4]. The collimator is a fixed beam, which does not rotate during treatment, which makes it difficult to change the irradiation field multiple times during treatment in terms of irradiation time the patient repositioning. Therefore, based on the current treatment protocol, the maximum number of overlapping irradiation fields was set to two. To fit the whole head within the irradiation field, collimator diameter was set to 18 cm.

**Fig. 2:**
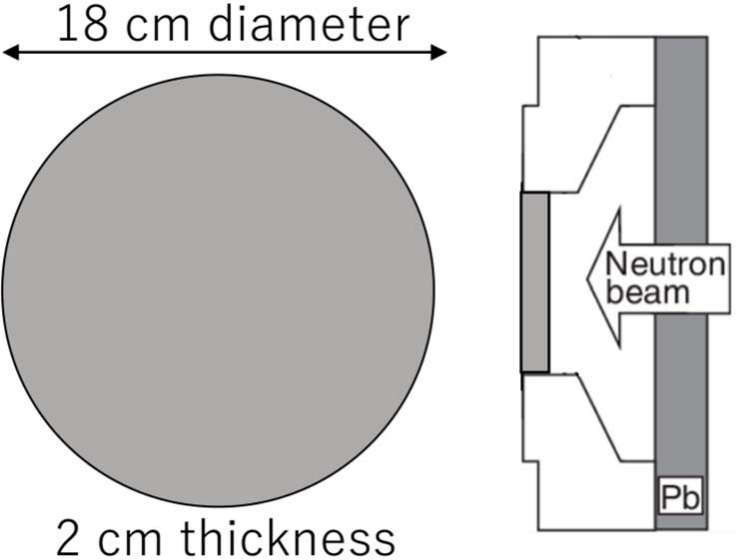
Schematic layout of IF-A.

The shape of the first intensity modulator was selected as to enhance the thermal neutron flux over the entire irradiation field. A PE disk with a thickness of 2 cm and diameter of 18 cm was chosen as the intensity modulator to enhance the thermal neutron flux at the tumor area [9]. This irradiation field with the PE disk is called irradiation field A (IF-A). Figure 2 shows a schematic diagram of IF-A.

**Fig. 3:**
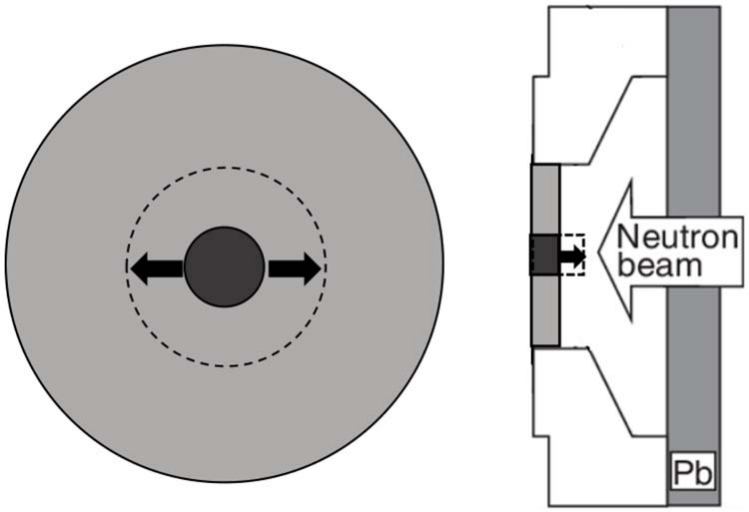
Schematic layout of intensity modulator shape design of IF-B.

The second intensity modulator was fabricated by combining PE and LiF-PE. A disk made of LiF-PE was inserted in the center of the 2 cm thick, 18 cm diameter disk used in IF-A. The diameter and thickness of this LiF-PE disk were varied to achieve higher thermal neutron flux at the edges relative to that at the center. The irradiation field with this intensity modulator is called the irradiation field B (IF-B). A schematic of the design of the intensity modulator shape in IF-B is shown in Fig. 3.

The diameter and thickness of the intensity modulator used in IF-B were determined to become the average thermal flux at evaluation points 5–7 in Fig. 1 (i.e. edge of the irradiation field) is higher compared to that of the evaluation points 1–4 (i.e. center of the irradiation field). The following ratio of average: ${R}_{\text{ave}}$ was used as the evaluation parameter.

$$ {R}_{\text{ave}}=\frac{\phi_{\text{edge}}}{\phi_{\text{center}}} $$

$$ {\phi}_{\text{edge}}=\frac{\phi_5+{\phi}_6+{\phi}_7}{3} $$

$$ {\phi}_{\text{center}}=\frac{\phi_1+{\phi}_2+{\phi}_3+{\phi}_4}{4} $$

${\phi}_{\text{edge}}$ is the average thermal neutron flux at evaluation points 5–7 at the edge of the irradiation field whereas ${\phi}_{\text{center}}$ is that at evaluation points 1–4 at the center of the irradiation field. A uniform thermal neutron irradiation field with a *R*_ave_ = 1 can be realized with the IF-B irradiation field alone. However the thermal neutron flux is $2.5\times{10}^8$ n/cm^2^/s, and the irradiation time becomes long. The clinical application is not possible. Therefore, it is necessary to irradiate IF-B to enhance the thermal neutron intensity at the edge while securing a sufficient thermal neutron flux with IF-A.

Here, as an evaluation index of IF-B, the condition where the *R*_ave_ was the highest was investigated so that the flux at the edges was higher than that at the center.

The ratio of irradiation time for IF-A and IF-B was determined to be the minimum value of thermal neutron flux at all evaluation points was more than $4.0\times{10}^8$ n/cm^2^/s corresponding to irradiation time of approximately 60 min to ensure uniformity. The uniformity index $u$ is defined as a measure of the uniformity of the thermal neutron flux using the following equation:

$$ u=\frac{\sum_1^7\left|100\times \left(1-\frac{\phi_{\text{i}}}{\phi_{\text{av}}}\right)\right|}{7} $$

$$ {\phi}_{\text{av}}=\frac{\phi_1+{\phi}_2+{\phi}_3+{\phi}_4+{\phi}_5+{\phi}_6+{\phi}_7}{7} $$

${\phi}_{\text{av}}$ is the average thermal neutron flux, and ${\phi}_{\text{i}}$ is the thermal neutron flux at each evaluation point.

### Irradiation test

**Fig. 4:**
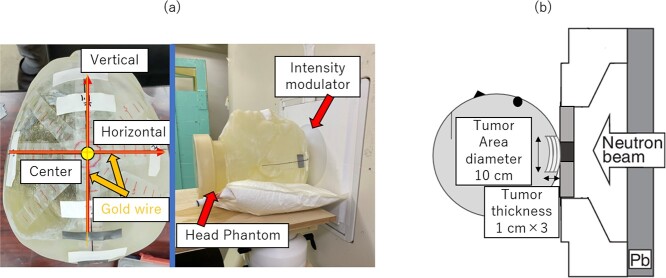
(a) Irradiation test and gold wire on the head phantom. (b) Schematic layout of SERA calculation.

Intensity modulators were fabricated, and irradiation tests were conducted using C-BENS. Figure 4 shows the irradiation test and head phantom with gold wire installed.

Gold wires were placed horizontally and vertically passing through the center of irradiation field. To confirm symmetry in the left–right direction, gold wires were placed in the left and right directions from the center of the irradiation field. The phantom was placed in front of the collimator of C-BENS, and IF-A and IF-B were irradiated. The center of irradiation point was shown in Fig. 4. The phantom was set up in close contact with the collimator surface. The phantom was placed with a laser marker installed in the irradiation chamber so that the point where the gold wire crosses coincides with the center of the irradiation field. In addition, the same procedure was repeated with Cd-covered gold wire. The thermal neutron flux at the phantom surface was derived by measuring induced activity of ^198^Au [8]. The installed gold wire was cut every 5 mm, and the thermal neutron flux derived each was used as the thermal neutron flux for each evaluation point. The experimentally measured thermal neutron flux was compared with the SERA calculation.

### Evaluation of tumor dose distribution

The uniformity of the thermal neutron flux was evaluated with intensity modulators by measurements and calculations. The relative biological effectiveness (RBE) equivalent dose to the tumor was determined. The irradiation conditions were as follows: normal skin dose - 15 Gy-eq; blood boron concentration - 24 ppm; T/B ratio was 1.0 for normal skin and 3.5 for the tumor. T/B ratio corresponds to the ratio of the boron concentration in the tumor to the blood boron concentration. The RBE value of the hydrogen dose was assumed at 2.4, nitrogen dose at 2.9, and gamma dose at 1.0 [4]. The compound biological effectiveness (CBE) values for the boron dose were set at 3.8 for tumors and 2.5 for normal skin. The dose to the tumor was calculated and the dose distribution was evaluated using dose volume histogram (DVH). The homogeneity index (HI) was used as an index of dose uniformity in tumors.

$$ HI=\frac{D_2-{D}_{98}}{D_{50}} $$

${D}_2$ , ${D}_{50}$, and ${D}_{98}$ are the doses (Gy-eq) at which 2%, 50%, and 98% volume of tumors is irradiated, respectively. The ideal value of HI is 0.

During treatment, blood boron concentrations can range from 12–37 ppm [4, 15]. This change in boron concentration affects the minimum tumor dose. Therefore, in this study, we calculated the minimum tumor dose using SERA when the blood boron concentration varied from to 10–40 ppm and evaluated the effect of variation in blood boron concentration on the irradiation method.

In addition, the thickness of the tumor may vary, and it may have spread deeper into the body. Therefore, we verified whether the intensity-modulated irradiation method is effective when the tumor is thicker. Parameters such as the minimum tumor dose and HI were calculated and evaluated from DVH, assuming that the thickness varied from 0.1 cm to 3 cm, while the tumor diameter was maintained at 10 cm. The schematic layout of a calculation model of SERA with increased tumor thickness was shown in Fig. 4(b).

## RESULTS

### Determination of the shape of the intensity modulators

**Fig. 5:**
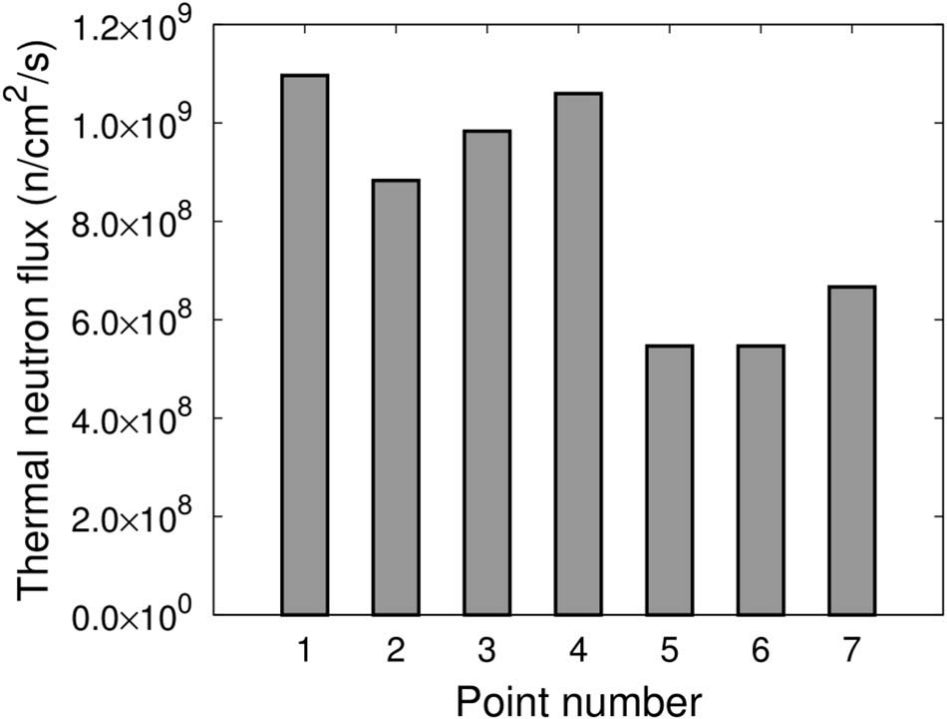
Thermal neutron flux in IF-A.

Figure 5 shows the calculated thermal neutron flux at each evaluation point in IF-A and the calculated thermal neutron flux distribution is high in the center of the irradiation field and low at the edge of IF-A.

**Fig. 6:**
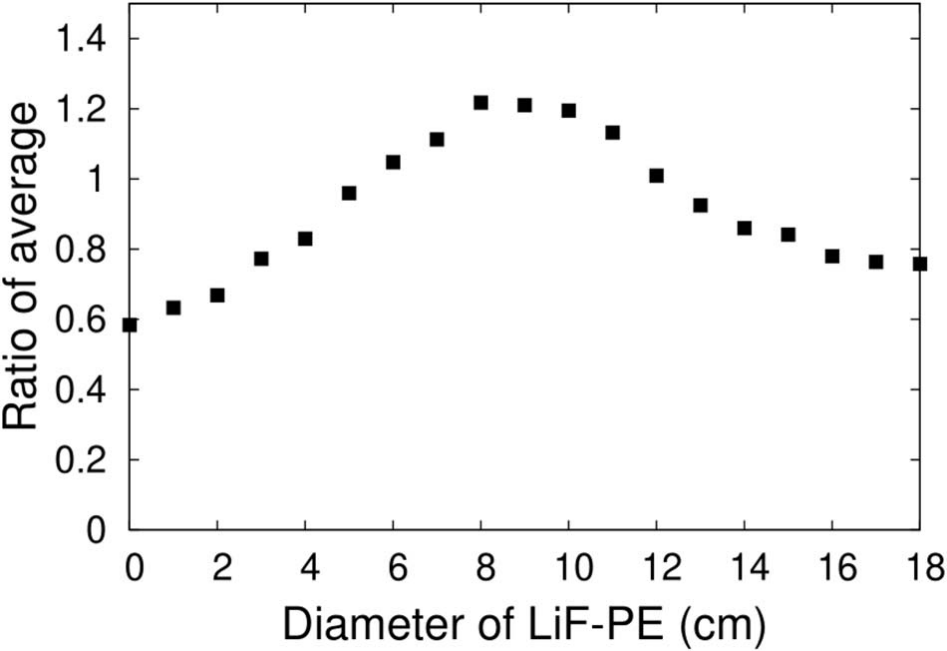
Ratio of average when the diameter of the LiF-PE disk is varied.

Figure 6 shows the ratio of average for varying diameters of the LiF-PE disk in IF-B. The calculated thermal neutron flux at the edge of the irradiation field was the highest when the diameter of the LiF-PE disk was 8 cm. Therefore, the diameter of the LiF-PE disk was set to 8 cm.

**Fig. 7:**
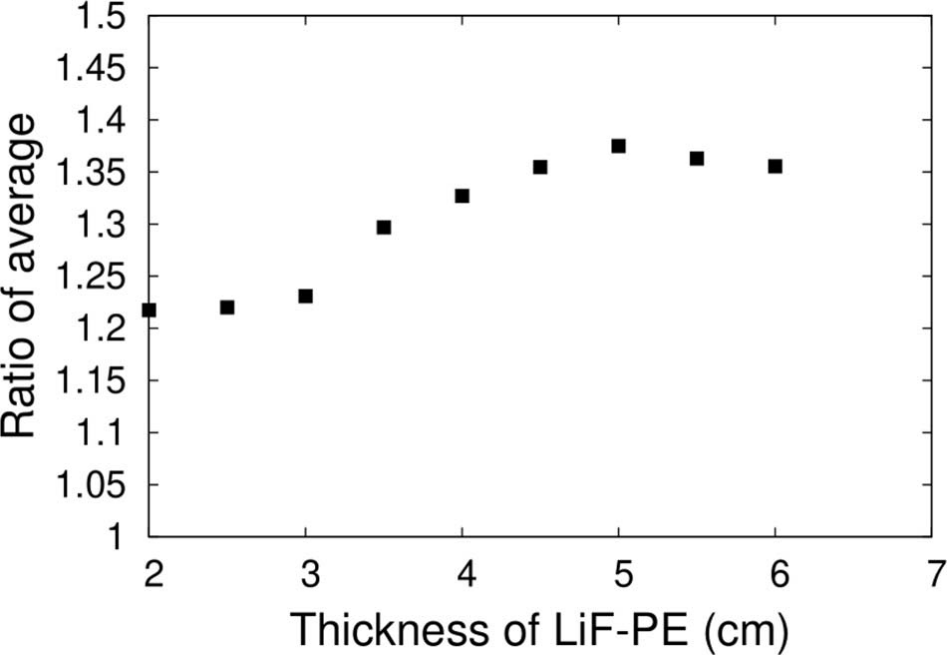
Ratio of average when the height of LiF-PE disk is varied.

The ratio of average when the thickness of the LiF-PE disk was varied is shown in Fig. 7. The calculated thermal neutron flux at the edge of the irradiation field was highest when the thickness of the LiF-PE disk was 5 cm. Therefore, the thickness of the LiF-PE disk was set to 5 cm.

**Fig. 8:**
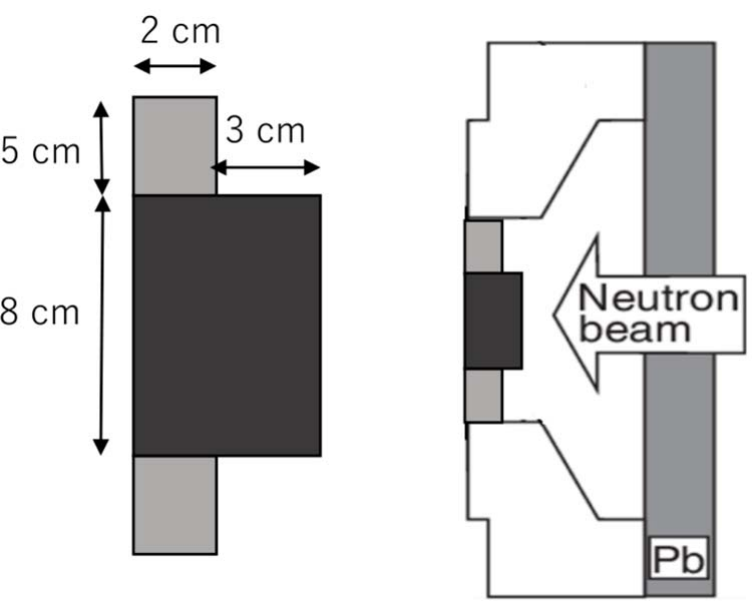
Schematic of IF-B.

Based on these results, the intensity modulator of IF-B was designed, as shown in Fig. 8.

**Fig. 9:**
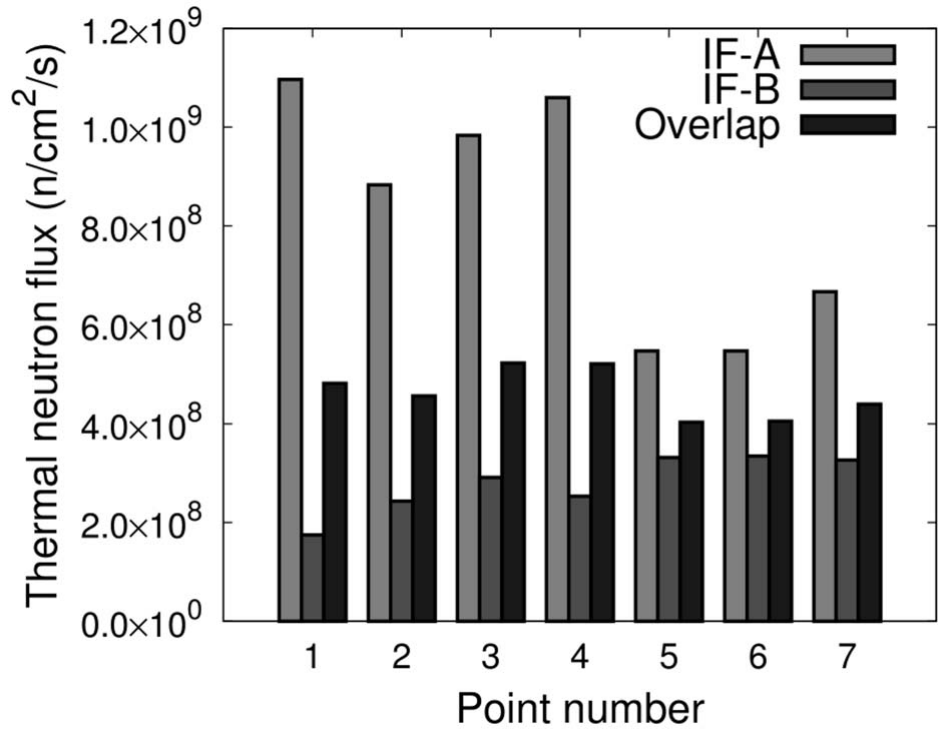
Thermal neutron flux when the ratio of irradiation time of IF-A and IF-B is 1:2.

Figure 9 shows the calculated thermal neutron flux for IF-B and when IF-A and IF-B overlapped with an irradiation time ratio of 1:2. The ‘Overlap’ flux is defined by the following equation. ${F}_{\text{IF}-\text{A}}$is the thermal neutron flux of IF-A, ${F}_{\text{IF}-\text{B}}$is the thermal neutron flux of IF-B.

$$ {F}_{\text{overlap}}=\frac{F_{\text{IF}-\text{A}}+2\times{F}_{\text{IF}-\text{B}}}{3} $$

According to Fig. 9, the distribution of the calculated thermal neutron flux in IF-B is relatively higher near the edges (evaluation points 5, 6, and 7) than at the center (evaluation points 1, 2, 3, and 4). Thus, a uniform thermal neutron flux can be achieved by overlapping IF-A and IF-B. Comparing the uniformity $u$, ${u}_{\text{A}}=24.8$ for IF-A, ${u}_{\text{B}}=17.1$ for IF-B, and ${u}_{\text{overlap}}=8.7$ when they were overlapping.

Therefore, it was revealed that a uniform thermal neutron flux distribution could be achieved by designing an appropriate intensity modulator and overlapping the irradiation fields.

### Irradiation test

**Fig. 10:**
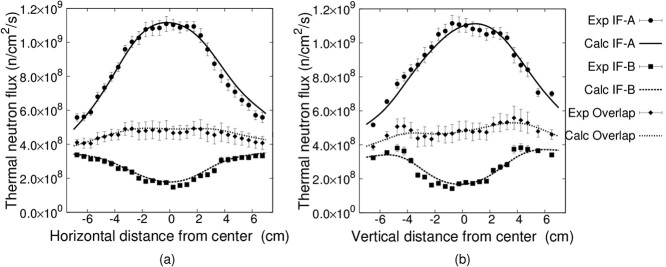
Thermal neutron flux distribution on the phantom surface (a) Horizontal direction (b) Vertical direction.

The intensity modulators for IF-A and IF-B were fabricated and irradiated. The measured thermal neutron fluxes and SERA calculations are shown in Fig. 10. A uniform thermal neutron flux distribution can be formed by overlapping the irradiation fields. In addition, the measured values were in good agreement with the values calculated by SERA. The distribution in the horizontal direction was symmetrical, owing to the horizontal uniformity of the head shape. However, the distribution in the vertical direction shows some variation owing to the vertical non-uniformity of the head shape. Moreover, the thermal neutron flux was obtained by cutting a single gold wire placed at the time of irradiation every 5 mm and measuring the activation reaction rate of each wire. This explains the variation in the thermal neutron flux at each location.

### Evaluation of tumor dose distribution

**Fig. 11:**
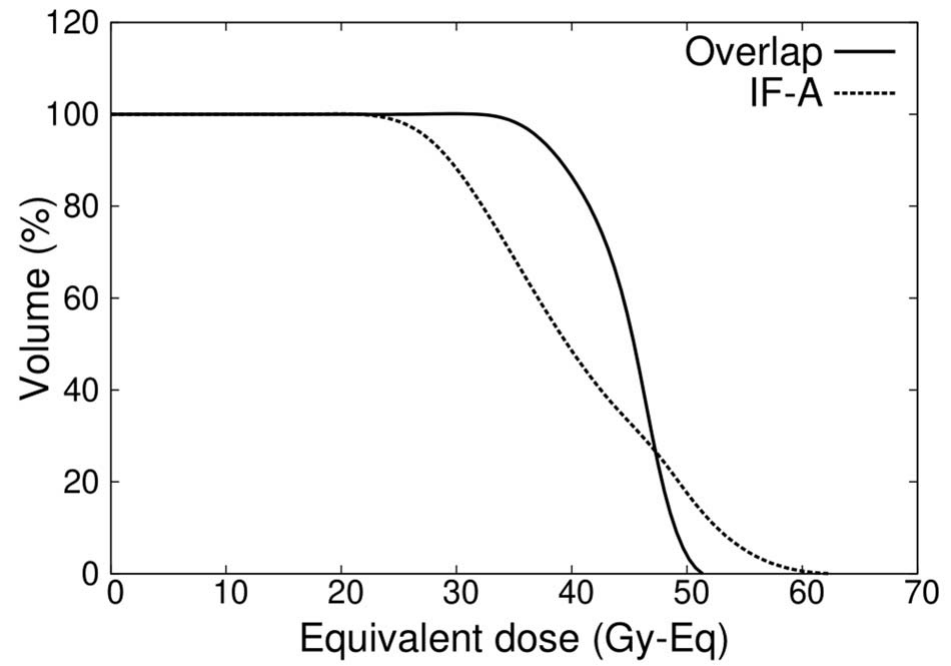
DVH in the tumor area.

The DVH in the tumor area is shown in Fig. 11 for IF-A and overlapping of IF-A and IF-B. The dose distribution in the tumor area is also improved by overlapping the irradiation fields. Table 1 shows the parameters of the irradiation.

**Table 1:** Comparison of parameters for whole-head irradiation

	Irradiation Time (min)	Minimum tumor dose (Gy-eq)	Homogeneity Index
IF-A + IF-B	70	27.4	0.36
IF-A	40	19.5	0.68

Table 1 shows that when irradiation fields overlap, the total irradiation time was 70 min, whereas the minimum tumor dose was 27.4 Gy-eq, which was higher than the tumor control dose of 20 Gy-eq [16, 17]. In contrast, for IF-A only, the irradiation time was shortened to 40 min, whereas the minimum tumor dose was lower than the control dose. A comparison of the HI values showed that the uniformity of the dose distribution was improved by overlapping the irradiation fields.

**Fig. 12:**
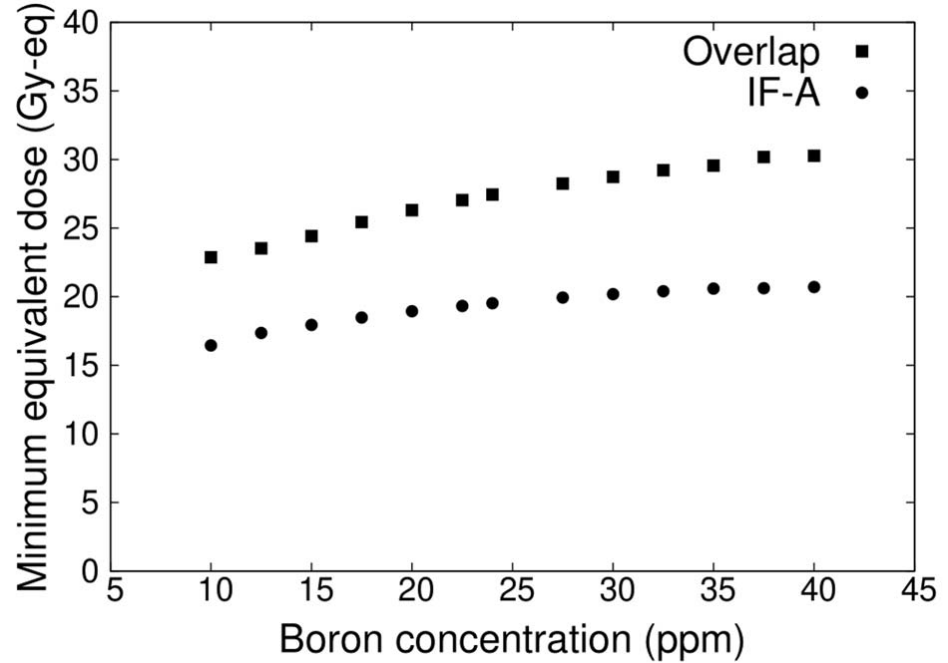
Minimum tumor dose at varying blood boron concentrations.

Figure 12 shows the minimum tumor dose for varying blood boron concentrations for the overlapping irradiation field, and IF-A only. The minimum tumor dose decreases as the blood boron concentration decreases in both, the IF-A field only and the overlapped irradiation fields. The tumor dose did not reach 20 Gy-eq for boron concentrations up to 40 ppm when irradiated with IF-A.

**Fig. 13:**
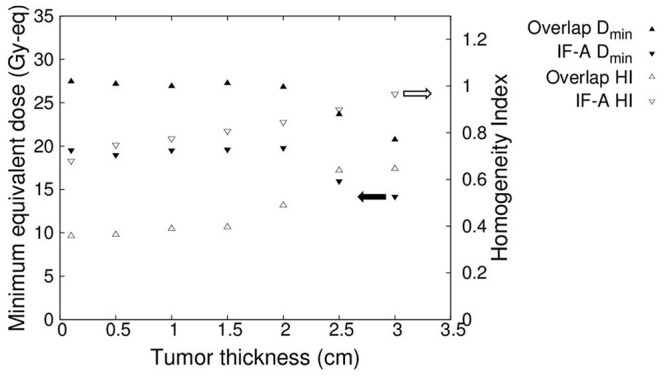
Minimum tumor dose and HI for varying tumor thickness.

Figure 13 shows the minimum tumor dose and HI in the case of overlapped irradiation fields and IF-A, when the tumor diameter was maintained at 10 cm and the thickness was varied. As shown in Fig. 13, as the tumor thickness increased, for IF-A, the minimum tumor dose was less than the control dose of 20 Gy-eq, making it difficult to administer a sufficient dose. In contrast, when the irradiation fields were overlapped, the minimum tumor dose exceeded the control dose of 20 Gy-eq for a tumor thickness of up to 3 cm. Therefore, it was found that the method of overlapping irradiation fields was effective even for a treatment depth of 3 cm. In both cases, the uniformity of the dose distribution was adversely affected as the tumor thickness increased.

For each tumor thickness SERA calculations were performed showing some variation in each case whereas the minimum tumor dose remained almost constant up to a thickness of 2 cm. This minimal dose corresponded to the tumor dose on the surface and at the edge. On the other hand, when the tumor thickness exceeds 2 cm, thermal neutrons are absorbed and decreased at a deep position, so that the deep position of the margin is minimized. As the thickness increases, the minimum dose decreases.

Owing to the thermal neutron flux distribution in the tumor, HI tends to increase as the tumor thickness increases.

## DISCUSSION

Previous studies have shown the possibility of using a bolus to treat superficial tumors with a diameter of 5 cm using an accelerator-based BNCT [8]. In Fig. 10, the experimental and calculated results show that the intensity-modulated irradiation method proposed in this study can produce a uniform thermal neutron flux and uniform dose distribution for a 10 cm diameter, 0.1 cm thick region at the top of the head. In contrast, for bolus-based treatments, a large bolus should be used for a wide range of tumors, whereas the lack of fit to the affected area in these cases causes uneven doses. On way to overcome this problem is to create a bolus, which snugly fits the affected area. However, such a specially made bolus is very costly and time-consuming to create. Furthermore, the reproducibility between the treatment planning CT and the actual treatment is an important issue in actual clinical practice. Thus, the method proposed in this study offers means to overcome all the aforementioned difficulties as it is applicable to a wide range of tumors at low cost and in a short time enabled by combining intensity modulators. Moreover, the developed irradiation method was found to be suitable for a wider region.

In addition, because the blood boron concentration at the time of treatment can only be measured immediately before irradiation, a tumor-controllable dose can be prescribed even when the blood boron concentration is lower than expected. When the irradiation fields overlapped, a minimum tumor dose of 20 Gy-eq or more was maintained, even when the boron concentration was 10 ppm.

A modulator installed in a collimator has been used to shift the peak of the thermal neutron flux in the body, demonstrating the feasibility of treating superficial tumors [9]. By comparing these studies, we can show that our proposed technique is adaptable to superficial tumors of a large area and thickness. If we choose an intensity modulator with a ratio of averages equal to 1, the thermal neutron flux average across the evaluation points will be very low. The D_min_ and HI were also better when the irradiation fields were overlapped. The overlapping irradiation fields raise the thermal neutron flux over the entire tumor region and improve the uniformity. This is mainly because the treatment with overlapping irradiation fields provides a better uniformity of the thermal neutron flux and dose distribution compared to the treatment with a single irradiation field.

The minimum dose for IF-A was below 20 Gy-eq because the edge of the irradiation field could not be irradiated with sufficient thermal neutrons. When the tumor thickness exceeded 2 cm, the minimum dose dropped sharply because thermal neutrons were absorbed, scattered, and attenuated in the body. Similarly, the overlapped irradiation fields attenuate thermal neutrons, but the overall dose that can be administered is large because the uniform thermal neutron flux distribution also makes the dose distribution uniform and improve the minimum dose; therefore, it is possible to treat tumors as thick as 3 cm.

The intensity-modulated irradiation method of accelerator-based BNCT proposed in this study is expected to be adaptable to various tumor shapes, sizes, thickness, and site, by changing the shape of the intensity modulator. In the future, we plan to automate this method and make it widely applicable. To adapt BNCT to the deep and wide tumors evaluated in this study, new techniques, such as multi-port irradiation with epithermal neutrons, will be necessary.

In addition, to apply this method, irradiation must be completed within one hour, and considering the time required to reposition the patient and the changing the modulators between fields, the neutron intensity of the accelerator-based neutron source may need to be further increased.

In conclusion, to develop an irradiation method for superficial tumors in accelerator-based BNCT, we developed an intensity-modulated irradiation approach by overlapping the irradiation fields using intensity modulators. The experimental results showed that a uniform thermal neutron flux distribution and dose distribution can be achieved over a 10 cm diameter area by overlapping the irradiation fields. Furthermore, it was found that tumor control was possible in the same area, up to a depth of approximately 3 cm. By applying this method, accelerator-based BNCT can be applied to tumors that are relatively shallow and spread over a wide area.

## Funding

This work was supported by Japan Science and Technology Agency (JST) Support for Pioneering Research Initiated by the Next Generation (SPRING), Grant Number JPMJSP2110.

## CONFLICT OF INTEREST

The authors declare that there are no conflicts of interest.
